# GT198 Is a Target of Oncology Drugs and Anticancer Herbs

**DOI:** 10.3389/froh.2021.679460

**Published:** 2021-06-11

**Authors:** Junfeng Pang, Jie Gao, Liyong Zhang, Nahid F. Mivechi, Lan Ko

**Affiliations:** ^1^Georgia Cancer Center, Medical College of Georgia, Augusta University, Augusta, GA, United States; ^2^Department of Clinical and Diagnostic Science, The University of Alabama at Birmingham, Birmingham, AL, United States; ^3^Department of Surgery, University of Pittsburgh, Pittsburgh, PA, United States; ^4^Research and Development, OnkoTarget, Augusta, GA, United States

**Keywords:** tumor angiogenesis, oncology drug target, anticancer herbs, GT198, oral cancer

## Abstract

Tumor angiogenesis is a hallmark of cancer. Therapeutic drug inhibitors targeting angiogenesis are clinically effective. We have previously identified GT198 (gene symbol *PSMC3IP*, also known as Hop2) as an oncoprotein that induces tumor angiogenesis in human cancers, including oral cancer. In this study, we show that the GT198 protein is a direct drug target of more than a dozen oncology drugs and several clinically successful anticancer herbs. GT198 is a DNA repair protein that binds to DNA. Using an *in vitro* DNA-binding assay, we tested the approved oncology drug set VII from the National Cancer Institute containing 129 oncology drugs. Identified GT198 inhibitors include but are not limited to mitoxantrone, doxorubicin, paclitaxel, etoposide, dactinomycin, and imatinib. Paclitaxel and etoposide have higher binding affinities, whereas doxorubicin has higher binding efficacy due to competitive inhibition. GT198 shares protein sequence homology with DNA topoisomerases, which are known drug targets, so that GT198 is likely a new drug target previously unrecognized. To seek more powerful GT198 inhibitors, we further tested several anticancer herbal extracts. The positive anticancer herbs with high affinity and high efficacy are all clinically successful ones, including allspice from Jamaica, *Gleditsia sinensis* or honey locust from China, and BIRM from Ecuador. Partial purification of allspice using an organic chemical approach demonstrated great feasibility of natural product purification, when the activity is monitored by the *in vitro* DNA-binding assay using GT198 as a target. Together, our study reveals GT198 as a new targeting mechanism for existing oncology drugs. The study also delivers an excellent drug target suitable for compound identification and natural product purification. In particular, this study opens an opportunity to rapidly identify drugs with high efficacy and low toxicity from nature.

## Introduction

Angiogenesis is a hallmark of many types of human solid tumors [1, 2]. Drugs that target to tumor angiogenesis are proven to have great therapeutic values [3–5]. Mounting evidence indicates that angiogenic blood vessels in tumor tissues are not normal counterparts; rather, they serve as the epicenters of tumor development [6–9]. In tumor tissues, angiogenic blood vessels could be malignant themselves [10].

Central to the control of angiogenesis is the pericyte [11]. Pericytes are perivascular cells initiating angiogenesis in response to stimuli [12]. Pericytes are stem or progenitor cells capable of differentiating into various cell lineages [13]. Normal pericytes may differentiate into new tissues during embryonic development or adult tissue repair [11–13]. However, this process is hijacked in tumors in which pericytes are angiogenic and malignant [14], and their differentiation is jeopardized. The angiogenic pericytes detach, migrate, and overgrow into undifferentiated tumor cells [10]. Tumor cells with migratory pericyte behavior have been previously observed [15]. Pericyte-derived tumor cells surrounding blood vessels are also described as vascular mimicry [6, 16, 17]. Multiple lines of evidence support that sustained tumor cell growth is replenished by cancerous stem cells [13], which would include angiogenic pericytes [11, 18].

To target tumor angiogenesis, a number of angiogenesis drug inhibitors have been developed [19, 20]. Many are kinase inhibitors or VEGF pathway inhibitors [21, 22], acting on blood vessel formations. Drug resistance may occur due to tumor vascular mimicry lacking endothelial cells [16], so that targeting to angiogenic pericytes could overcome drug resistance and suppress tumor angiogenesis more specifically. We and others have previously identified a DNA repair oncoprotein called GT198 (gene symbol *PSMC3IP*, alias Hop2) [10, 23–25]. The GT198 protein is overexpressed in angiogenic pericytes in tumors but not in quiescent normal pericytes [10]. In this study, we show that GT198 protein is an excellent drug target, inhibited by a panel of approved oncology chemotherapeutic drugs as well as several anticancer herbs known to be effective in human cancer treatment.

The GT198 protein is originally characterized as a transcriptional coactivator [23, 26]. The human *GT198* gene carries germline mutations in early-onset breast and ovarian cancer families [27, 28], in familial ovarian disease [29], and insufficiencies [30–32]. Somatic mutations in *GT198* are prevalent and recurrent in the tumor microenvironment of sporadic cancers [27, 33]. Importantly, *GT198* mutations lead to its protein overexpression, which can be detected in angiogenic pericytes and their descendent vascular smooth muscle cell lineage, such as myoepithelial cells and adipocytes in breast cancer [34], theca cells in ovarian cancer [35], and myofibroblasts in prostate and bladder cancers [36]. GT198-expressing pericytes have also been found in oral cancers as well as in multiple other solid tumors [10] and in mouse tumors [37]. Abnormal angiogenic pericytes in the microenvironment appear to be the origin of human cancer development.

The role of GT198 in pericytes may be associated with its expression and function in stem cells [10, 33]. Overall GT198 expression resembles cancer-testis antigens with high levels in the embryo, testis, cancer, and with low levels in normal adult tissues [10]. Normal stem cell differentiation requires a switch from functionally counteracting te GT198 splice variant to its wild type [33]. In human tumors, identified somatic mutations generate variants similar to the normal splice variants so that stem cell differentiation could be jeopardized [33]. Indeed, the angiogenic pericytes in oral tumor stroma produce undifferentiated cells [10].

GT198 is a small DNA-binding protein dimer, containing 217 amino acids in its monomer [23, 33]. The GT198 protein includes an N-terminal domain, a leucine zipper dimerization domain, a DNA-binding domain capable of binding to either single- or double-stranded DNA [24, 33], and a C-terminal auto-inhibitory domain [33]. Many biochemical studies published using a GT198 alias name Hop2 demonstrate that mammalian GT198 is a critical DNA repair factor stimulating homologous DNA recombination and regulating meiosis [24, 38, 39]. The mounting evidence collectively puts GT198 as a master nuclear controller with central importance. The processes of transcriptional activation, recombination in DNA repair, and pairing of homologous chromosomes in meiosis, all require the opening and binding of DNA strands. Thus, the DNA-binding is a critical activity of GT198. Through detecting its DNA-binding activity, GT198 inhibitors can be tested.

In this report, we extend our previous observations [10], and further show angiogenic pericytes expressing GT198 in human oral cancer. To test whether GT198 could be a target of angiogenesis inhibitors, we screened 129 clinical oncology drugs derived from the National Cancer Institute (NCI) using an *in vitro* DNA-binding assay of GT198. Surprisingly, we found that a panel of chemotherapeutic drugs, including angiogenesis inhibitors, such as doxorubicin, mitoxantrone, paclitaxel, etoposide, and gleevec, are also GT198 inhibitors. GT198 is likely a hidden drug target previously unrecognized. To further seek powerful GT198 inhibitors, we tested a number of anticancer herbs with historical success in human cancer treatment. We have identified several positive herbs including Jamaican allspice, Chinese *Gleditsia Sinensis*, and Ecuadorean BIRM, which directly inhibit GT198 with high affinity and efficacy. Partial purification of allspice using methods in organic chemistry confirmed great feasibility of natural product purification monitored by inhibiting of GT198 activity. Together, our finding reveals that GT198 is a hidden target of many existing oncology drugs and anticancer herbs. GT198 can be served as a target for future chemical compound drug identification and also natural product purification because a target is now available.

## Results

### Angiogenic GT198^+^ Pericytes Give Rise to Human Oral Cancer

**Figure 1:**
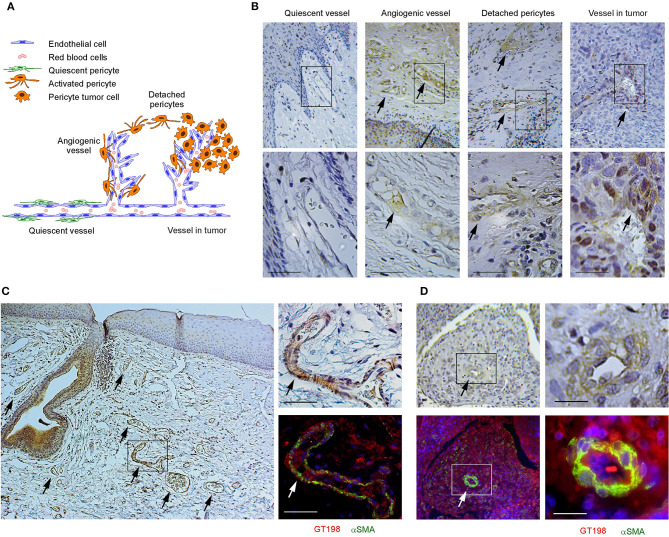
GT198^+^ angiogenic blood vessels in human oral cancers. **(A)** A hypothetic model comparing pericytes in quiescent vessel in green and in angiogenic and tumor enclosed vessels in orange. **(B)** Immunohistochemical staining of GT198 in human oral cancer. Pericytes in quiescent vessel are GT198^−^. Angiogenic pericytes, detached pericytes, and pericytes in a tumor are GT198^+^. The tumor enclosed vessel is functional with red blood cells inside. Boxed areas are enlarged below. **(C)** In an epithelial oral cancer, multiple GT198^+^ angiogenic vessels are present around the GT198^+^ epithelia in tumor development. An adjacent section is immunofluorescent double-stained with GT198 in red and αSMA in green and counterstained with DAPI in blue. **(D)** An oral tumor enclosing a vessel from the same patient is double-stained with GT198 and αSMA showing a vessel at tumor center. Boxed areas are enlarged at the right. Extended view is shown in Supplementary Figure 1. Arrows indicate GT198^+^ pericytes. Immunohistochemistry sections are counterstained with hematoxylin. Scale bars = 100 μm.

We have previously identified GT198-expressing angiogenic blood vessels in precursor lesions of various human solid tumors, including oral cancer [10, 34–36]. In agreement with our previous finding in human oral cancers, GT198 protein is a clear marker to reveal angiogenic blood vessels in the tumor microenvironment (Figures 1A,B). When oral cancer pathology slides are examined by immunohistochemistry of GT198, we observed that the earliest lesions are GT198^+^ pericytes, which appear to differentiate into multiple types of descendent cells. Initially, GT198^+^ pericytes are present in small clusters of angiogenic vessels while the surrounding quiescent vessels have GT198^−^ pericytes (Figure 1B). Next, the cytoplasmic volume of GT198^+^ pericytes increases so that the capillary wall abnormally thickens (Figure 1B). Further, GT198^+^ pericytes detach from vessels becoming nodules in tissue stroma. At this stage, GT198^+^ fibroblasts in stroma also increase. Then, the pericyte nodule proliferates into a tumor mass often enclosing a blood vessel at its center (Figure 1B). This type of blood vessel in a tumor is still functional with red blood cells in its cavity, which is consistent with the observations from others that the tumor produces its own vessels [6, 16]. It is important to note that classically described tumor development from epithelial basal layers can also be observed. However, in that case, the affected epithelia are always surrounded by GT198^+^ angiogenic blood vessels, which carry the vascular smooth muscle marker αSMA (Figure 1C). The affected epithelial cells are also GT198^+^ compared with nearby normal epithelia, which are GT198^−^ (Figure 1C). Because pericyte stem cells may replenish progenitor cells in the basal layer of the epithelium, it is possible that epithelium- and stromal nodule–derived oral tumors are both originated from angiogenic pericytes. The concepts of tumor initiation from epithelia vs. from angiogenic vessels may now be reconciled when using GT198 protein as a pericyte marker. Tumor initiation from vessels is further supported by scattered GT198^+^αSMA^+^ cells found within the tumors that enclose vessels (Figure 1D and Supplementary Figure 1). Angiogenic vessels appear to develop into early stage tumors and later disintegrate when tumors mature or advance. In agreement with our previous study [10], the evidence collectively suggests that angiogenic pericytes give rise to tumors in human oral cancer.

### Oncology Drugs Including Doxorubicin and Paclitaxel Are GT198 Inhibitors

**Figure 2:**
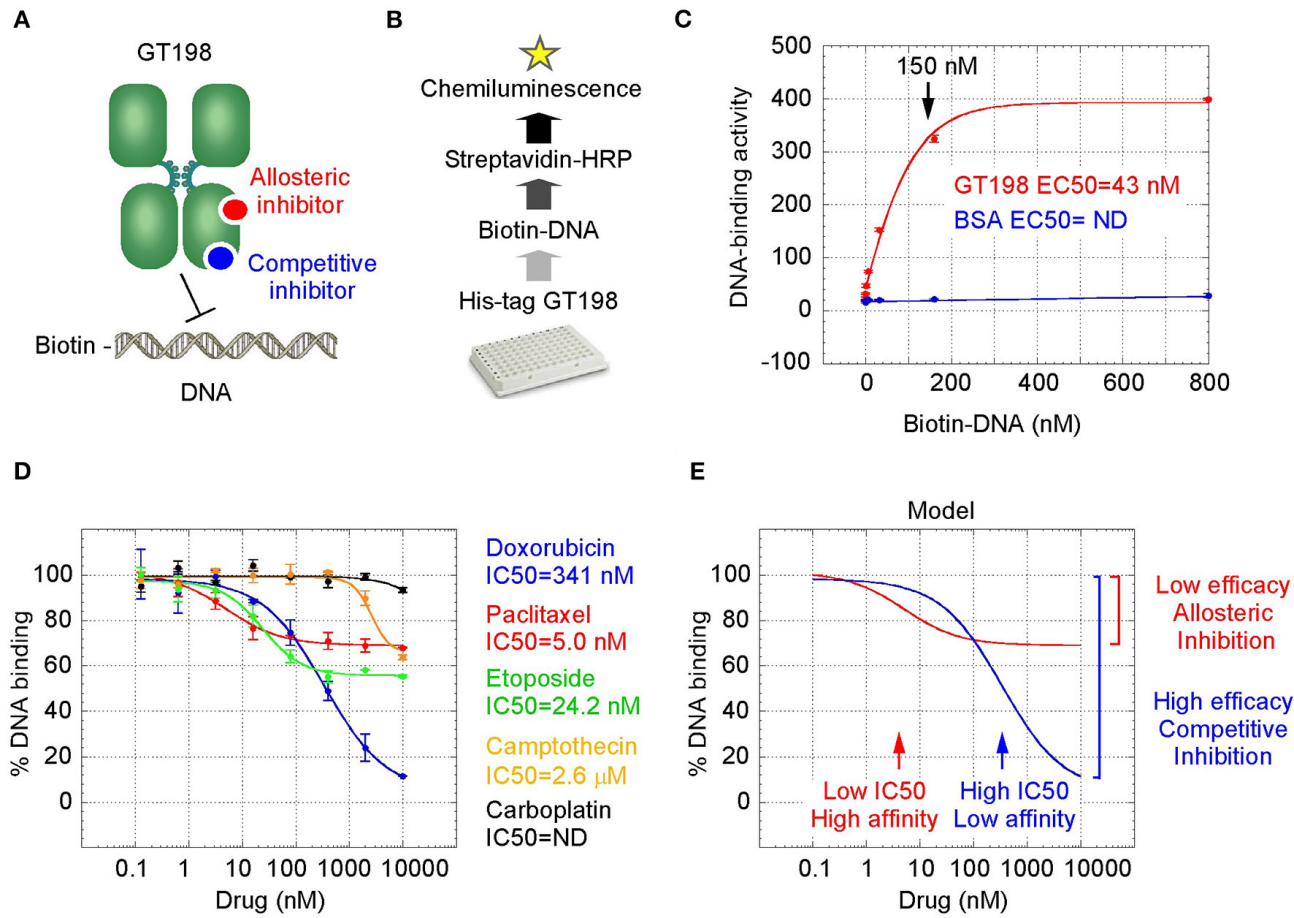
GT198 is a target of oncology drugs. **(A)** A model of allosteric or competitive drug inhibition of the DNA binding of GT198 dimer. **(B)** A flowchart of the DNA-binding assay. **(C)** The increasing concentrations of biotinylated DNA bind to GT198 at EC50 = 43 nM using BSA as a negative control. **(D)** Inhibition of DNA binding to GT198 by increasing concentrations of clinical drugs as indicated. IC_50_s are measured by sigmoidal curve fit. Data represent mean ± s.e.m of duplicate experiments. **(E)** A graph model comparing binding affinity and efficacy.

GT198 is a specific marker for tumor angiogenesis. To test the idea that GT198 inhibitors would have an anticancer effect, we first selected clinically successful oncology drugs for analysis. In order to measure GT198 activity *in vitro* for direct inhibition, a DNA-binding assay was developed in which recombinant GT198 proteins are coated on 96-well plates and assayed for the binding of biotin-labeled DNA in the presence of drug inhibitors. Bound biotinylated DNA can be detected by streptavidin-horseradish peroxidase (HRP) conjugate and chemiluminescence (Figures 2A,B). The binding affinity between DNA and GT198 was first tested to be EC_50_ = 43 nM using BSA as a negative control (Figure 2C). This result revealed a DNA concentration before the binding saturation as approximately 150 nM (Figure 2C), an optimal DNA concentration to allow both maximal signal detection and efficient drug competition in subsequent analyses.

A number of chemotherapy drugs were then tested for GT198 inhibition by measuring the IC_50_, half maximal inhibitory concentration under serial diluted concentrations of drugs and in the presence of 150 nM DNA. Surprisingly, we identified many positive GT198 inhibitors. Doxorubicin has low affinity (IC_50_ = 341 nM) but high efficacy for inhibition, and paclitaxel (IC_50_= 5.0 nM) and etoposide (IC_50_=24.2 nM) have high affinities and poor efficacies (Figures 2D,E). Camptothecin has poor affinity (IC_50_= 2.06 μM), and carboplatin does not inhibit GT198 (Figure 2D). This is the first time to cross-compare binding characteristics of these drugs *in vitro* using their common target. The finding may be clinically relevant because higher dose tolerance with low toxicity in paclitaxel or etoposide reflects their high affinities [40, 41], potent doxorubicin reflects its high efficacy albeit toxicity [42], and the clinical weakness of camptothecin in contrast to highly successful paclitaxel may be due to its very poor affinity [43, 44].

**Figure 3:**
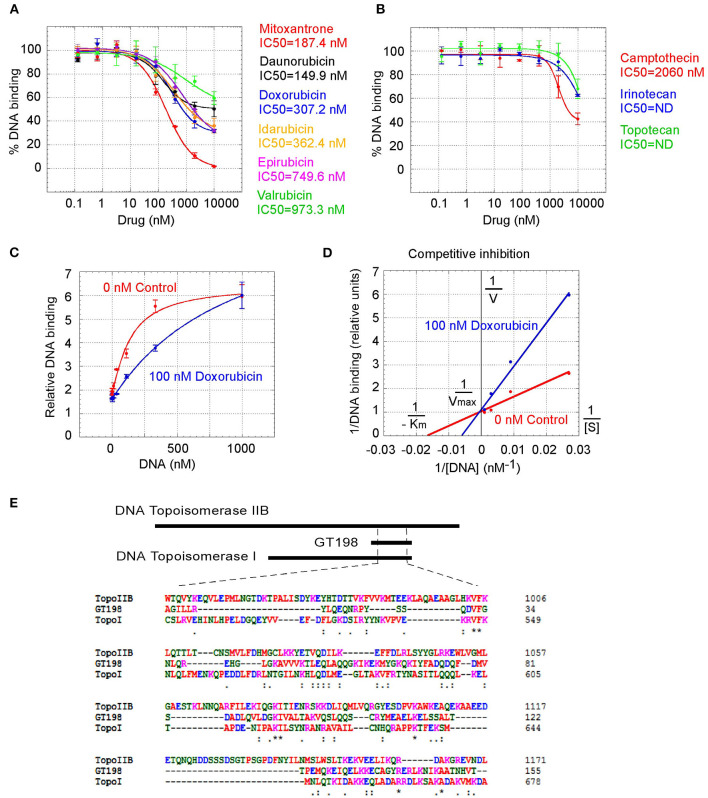
Competitive inhibition of GT198 by doxorubicin. **(A,B)** Inhibition of the DNA binding to GT198 under increasing concentrations of doxorubicin analogs and camptothecin analogs. Their IC_50_s are as indicated. **(C)** Increasing concentrations of biotinylated DNA bind to GT198 in the presence (blue) or absence (red) of 100 nM doxorubicin. Doxorubicin as a competitor shifts the binding curve to the right. **(D)** Double reciprocal plot using the means of high-concentration data points (37, 111, 333, 1,000 nM) from the panel C. The constant Vmax and increased Km with doxorubicin indicate competitive inhibition. **(E)** Primary sequence homology among human GT198 and DNA topoisomerase I and IIB (Clustal Omega). Asterisks denote identical residues and dots denote homologous residues. Full-length alignment is shown in Supplementary Figure 2.

GT198 being a genuine drug target became more evident when the similarities among drug analogs were found. Among doxorubicin analogs, mitoxantrone (IC_50_ = 187.4 nM) and daunorubicin (IC_50_ = 149.9 nM) showed better affinities than idarubicin (IC_50_ =362.4 nM), epirubicin (IC_50_ = 749.6 nM), and valrubicin (IC_50_ = 973.3 nM) (Figure 3A). Mitoxantrone has the highest efficacy leading to an almost complete inhibition. However, their binding affinities are all within the hundred nanomolar range. In contrast, camptothecin and its analogs irinotecan and topotecan have very poor affinities or fail to inhibit GT198 (Figure 3B).

We further found that doxorubicin is a competitive inhibitor of GT198 (Figures 3C,D). The binding of GT198 by doxorubicin competed with increasing concentrations of DNA (Figure 3C). Double-reciprocal plot analysis of the same data revealed competitive binding by DNA and doxorubicin (Figure 3D). The constant Vmax with increased Km in the presence of 100 nM doxorubicin vs. 0 nM control suggested that doxorubicin binds to the same site on GT198 as DNA binds. Thus, it directly blocks the DNA-binding site of GT198 (model in Figure 2A). As a competitive inhibitor, doxorubicin exerts high efficacy (model in Figure 2E). In contrast, paclitaxel was found to be an allosteric inhibitor or noncompetitive inhibitor in a separate study [45]. The allosteric inhibition may lead to rather incomplete inhibition, resulting poorer binding efficacy (Figures 2A–D). Our results provide explanations that doxorubicin is a more potent drug due to high efficacy, and paclitaxel is less potent but more sensitive due to higher affinity. Higher affinity is linked to low toxicity because lower drug concentration is sufficient.

It was initially unexpected but later no longer surprising for many drugs found to be GT198 inhibitors because GT198 shares protein sequence homology with DNA topoisomerases I and II (Figure 3E and Supplementary Figure 2). DNA topoisomerases are previously known targets of analogs of doxorubicin, etoposide, and camptothecin [46–48]. Like GT198, DNA topoisomerases are DNA-binding proteins participating in transcription and DNA recombination. Thus, the sequence homology further validates GT198 as a previously unrecognized target of topoisomerases inhibitors.

### Identification of GT198 Inhibitors From NCI Oncology Drugs Set VII

**Figure 4:**
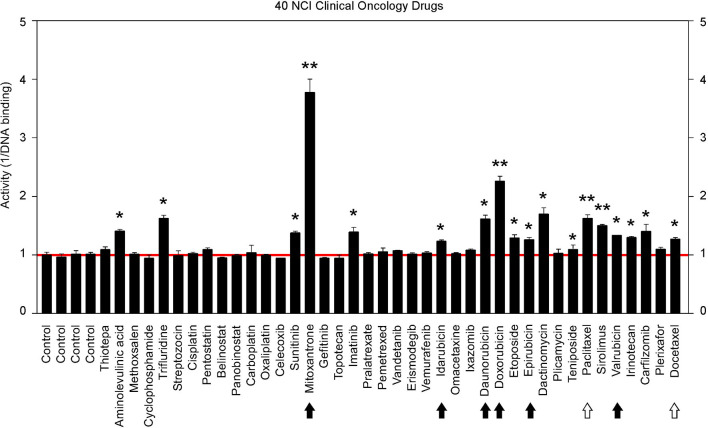
Clinical oncology drugs inhibit GT198. Clinical oncology drugs were tested by the DNA binding assay. The drug activities of GT198 inhibition are shown as the relative reciprocal amounts of DNA binding in a bar graph. Black arrows indicate doxorubicin analogs. White arrows indicate paclitaxel analogs. Stars indicate statistically significant activities. Selected 40 oncology drugs are shown, and the complete list is in Supplementary Table 1.

The above findings prompted us to further test additional oncology drugs as GT198 inhibitors. Using the DNA-binding assay above, we screened 129 oncology drugs from the Approved Oncology Drugs Set VII derived from the NCI (Table 1 and Supplementary Table 1). The selected 40 drugs are shown for comparison (Figure 4), and IC_50_ values and efficacies were only analyzed in a number of positive inhibitors (Table 1). Identified GT198 inhibitors include doxorubicin family analogs, paclitaxel and docetaxel; etoposide and teniposide. In addition, positive inhibitors also include dactinomycin, carfilzomib, sirolimus (rapamycin), imatinib (Gleevec), sunitinib, trifluridine, and aminolevulinic acid (Figure 4 and Table 1). Celastrol, which was not from the NCI drug collection, was also found to be positive (Table 1). Many drugs are negatives, including platinum inhibitors, methotrexate, and vincristine. Of all the drugs tested as GT198 inhibitors, mitoxantrone has the highest efficacy, and paclitaxel has the best affinity (Table 1) [45].

**Table 1:** Summary of clinical oncology drugs tested for GT198 inhibition.

**Drug name**	**IC_**50**_**	**Efficacy**	**Previous mechanism of action**
Mitoxantrone	187 nM	90%	Topoisomerase
Daunorubicin	149 nM	48%	Topoisomerase
Doxorubicin	307 nM	61%	Topoisomerase
Idarubicin	362 nM	56%	Topoisomerase
Epirubicin	749 nM	50%	Topoisomerase
Valrubicin	973 nM	26%	Topoisomerase
Paclitaxel (Taxol)	5 nM	32%	Tubulin
Docetaxel	–	Active	Tubulin
Cabazitaxel	–	Inactive	Tubulin
Vincristine	–	Inactive	Tubulin
Etoposide	24 nM	42%	Topoisomerase
Teniposide	–	Active	Topoisomerase
Camptothecin	2,060 nM	30%	Topoisomerase
Topotecan	>2,000 nM	4%	Topoisomerase
Irinotecan	>2,000 nM	10%	Topoisomerase
Dactinomycin	–	Active	Topoisomerase
Celastrol	350 nM	30%	Broad spectrum
Carfilzomib	–	Active	Proteasome
Sirolimus (Rapamycin)	–	Active	mTOR
Imatinib (Gleevec)	–	Active	Tyrosine kinase
Sunitinib	–	Active	Tyrosine kinase
Trifluridine	–	Active	Nucleoside DNA inhibitor
Aminolevulinic acid	–	Active	Photodynamic
Carboplatin	–	Inactive	Platinum-based DNA inhibitor
Cisplatin	–	Inactive	Platinum-based DNA inhibitor

*Drugs tested with IC_50_ are shown with percent efficacy measured at 2 μM. Drugs not measured with IC_50_ are shown as active or inactive. Hyphens denote IC_50_ unavailable*.

It is important to note that many drugs are well-characterized with other mechanism of actions in the history. For example, Gleevec is an Abl tyrosine kinase inhibitor. However, during traditional drug development, there are multiple selection procedures from hit to lead and further to candidate. Often, the later steps require cell and animal testing whereas the GT198 protein is a prominent cytotoxic target inducing apoptosis [33] and is also overexpressed in many mouse tumor models [10, 37]. Therefore, Gleevec may inhibit both Abl tyrosine kinase and GT198 *in vivo*, but this was not revealed until this study. Similarly, some other GT198 inhibitors identified here may also have more than one *in vivo* target.

### Anticancer Herbs Inhibit GT198

**Figure 5:**
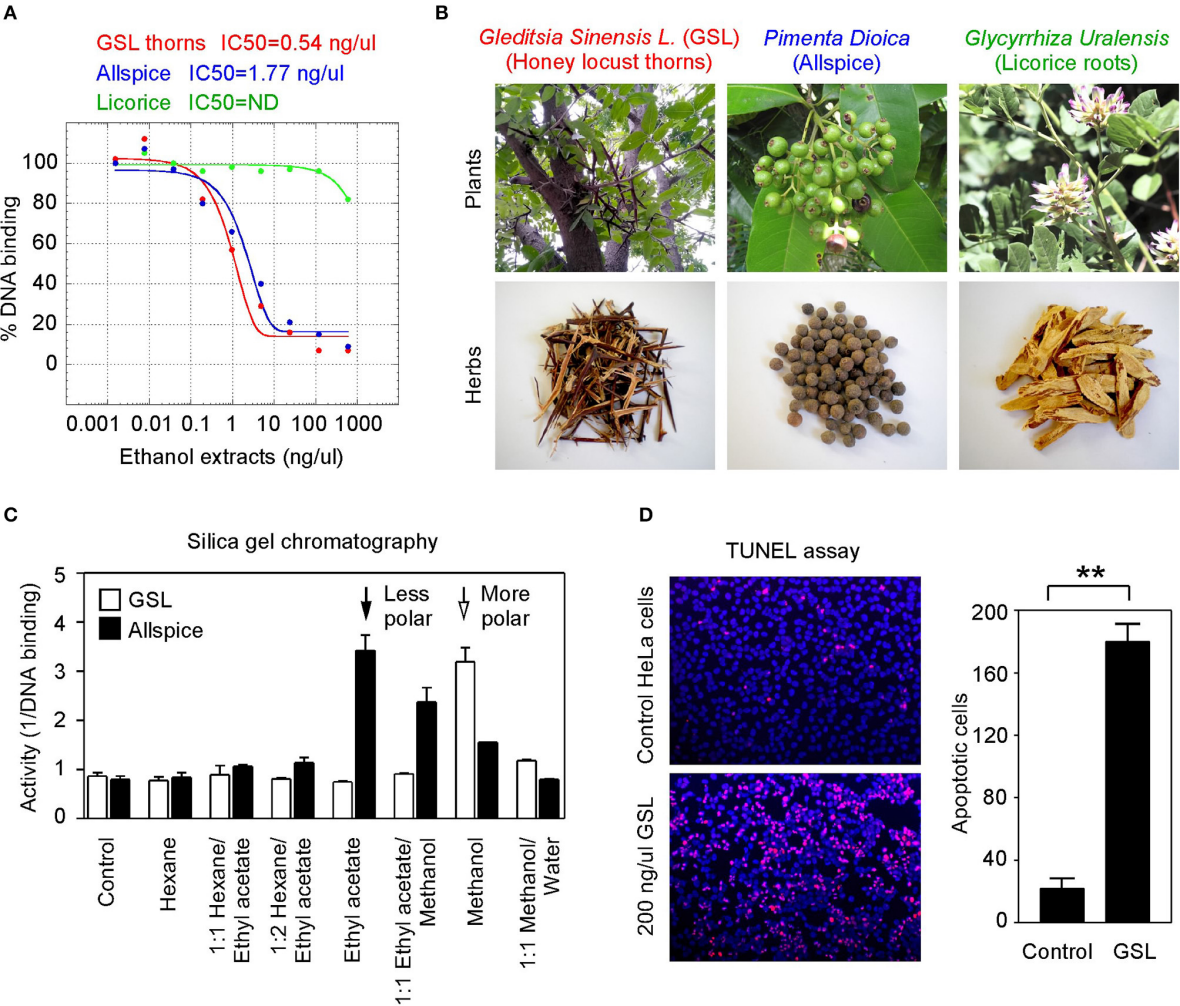
Anticancer herbs inhibit GT198. **(A)** The thorn needles of GSL and berries of allspice extracts inhibit the DNA-binding activity of GT198. Licorice is a negative control. IC_50_s are as indicated. **(B)** Photographs of the plants and their corresponding herbs used in panel A. **(C)** Silica gel chromatography of GSL and allspice. Arrows indicate their most active constituents with different polarities when eluted from nonpolar to polar solvents. **(D)** TUNEL assay for apoptotic activity of GSL in HeLa cells. Quantitative representation of positive cell numbers in microscopic views is shown on the right. ^**^P < 0.01.

To seek more potent GT198 inhibitors with both high affinity and high efficacy, we looked into herbs having historical success in the treatment of human cancers. Detailed rationales for each herb selection are described in the Discussion section. The two most promising herbs with a long history of cancer treatment, allspice (*Pimenta dioica*) native to Jamaica and honey locust thorns (*Gleditsia sinensis L.*, GSL) native to China, were extracted by ethanol and tested by the DNA-binding assay. Both allspice (IC_50_ = 1.77 ng/μl, efficacy = 86%) and GSL (IC_50_ = 0.54 ng/μl, efficacy = 92%) showed excellent affinities and efficacies compared with licorice root, which was used as a negative control (Figures 5A,B). Using silica gel chromatography to analyze eluting profiles for polarity, we found that the active ingredients from two plants have distinct polarity (Figure 5C). The active ingredient in GSL is more polar than that in allspice, suggesting the presence of two distinct drug inhibitors that can later be purified. When the GSL extract was tested by a TUNEL assay for apoptosis on HeLa cells, significant apoptotic activity was detected (Figure 5D). This result confirmed the presence of cytotoxic ingredient in the GSL extract. Allspice has been previously shown to be apoptotic [49].

**Figure 6:**
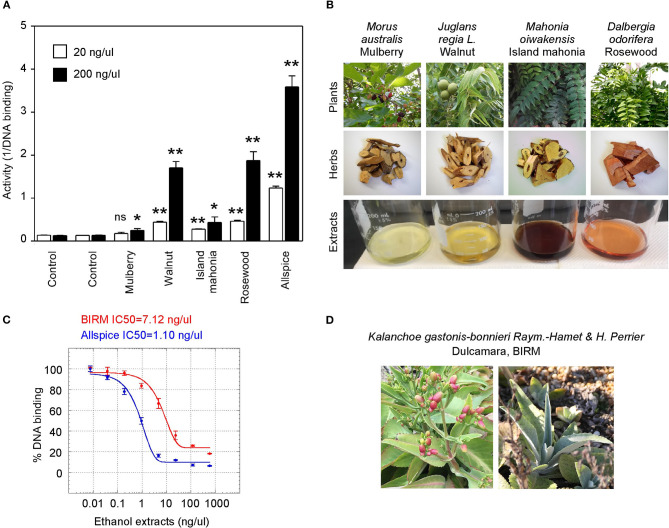
Commercial herbal medicines inhibit GT198. **(A)** Ethanol extracts from four tree branches inhibit the DNA-binding of GT198. The four trees are mulberry, walnut, island mahonia, and rosewood from a Taiwanese anticancer remedy. Allspice is a positive control. **(B)** Photographs of the four plants, their corresponding herbs, and ethanol extracts assayed in panel A. **(C)** BIRM, an Ecuador product of dulcamara root extract, inhibits the DNA-binding activity of GT198. Allspice as a positive control. IC_50_s are indicated. **(D)** Photographs of dulcamara plant. ^*^P < 0.05 and ^**^P < 0.01

We also tested a set of anticancer herbs originating from Taiwan containing branches of four trees (Figures 6A,B). The mix of four crude herbs is an anticancer remedy in Taiwan with extensive cancer treatment testimonies. These trees are mulberry (*Morus australis*), walnut (*Juglans regia L.)*, island mahonia (*Mahonia oiwakensis*), and rosewood (*Dalbergia odorifera*). When individually tested, we found that all four extracts positively inhibit GT198 although their activities are much lower than allspice as a positive control under the same concentrations (Figure 6A). All four herbs are commonly used in Asia, and their combinations may exert higher potency albeit low activity in each herb.

Another tested herb is a commercially available online health product from Ecuador called BIRM. BIRM stands for biological immune response modulator. BIRM is an aqueous extract of dried roots of the Ecuadorian plant dulcamara (*Kalanchoe gastonis-bonnieri Raym*). The BIRM extract showed high efficacy as well as high affinity (IC_50_ = 7.12 ng/μl) in inhibiting GT198 (Figures 6C,D). In addition to human cancer treatment testimonies, research studies have previously shown that BIRM is effective for prostate cancer by regulating androgen receptor in cellular and animal models [50, 51]. The current study first pinpoints that GT198 is a direct target of BIRM, while GT198 is also an androgen receptor coactivator [23].

Very interestingly, most identified anti-GT198 anticancer herbs are also herbs known to be anti-infection (Table 2). Online testimonies in the treatment of viral and bacterial infections by allspice and BIRM are extensive. GSL has also been shown to be anti-HIV [52] and was used for COVID-19 in the 2020 pandemic in Asia. Because angiogenesis is activated by both acute inflammation in infection and chronic inflammation in cancer, GT198 inhibitors suppressing angiogenesis could be a subset of dual effective anticancer and anti-infective drugs.

**Table 2:** Summary of herbs tested for GT198 inhibition.

**Name (Other name)**	**Activity**	**Anticancer**	**Anti-Infection**	**Plant origin**
***Latin name***	**IC_**50**_, Efficacy**			
Chinese honey locust (ZaoJiaoCi)	0.54 ng/μl, 92%	+++	++	China, Korea
*Gleditsia sinensis L*. (GSL)				
Allspice	1.77 ng/μl, 86%	+++	+++	Jamaica
*Pimenta dioica*				
BIRM (Dulcamara)	7.12 ng/μl, 74%	+++	+++	Ecuador
*Kalanchoe gastonis-bonnieri*				
Mulberry tree (SangZhi)	Weakly positive	+	++	China, Europe
*Morus australis*				
Walnut tree (HeTaoZhi)	Positive	++	+	Europe, Asia
*Juglans regia L*.				
Island mahonia (ShiDaGongLao)	Positive	+++	+++	Taiwan
*Mahonia oiwakensis*				
Rosewood (JiangXiang)	Positive	+++	+	India, S. Asia
*Dalbergia odorifera T. Chen*				
Liquorice (GanCao)	Negative	–	+	China
*Glycyrrhiza uralensis Fisch*				
Garden mum (JuHua)	Negative	–	+	Japan, China
*Chrysanthemum x morifolium*				

*Efficacies of GT198 inhibition are measured with 120 ng/μl of herbal ethanol extracts. Clinical evidence of anticancer and anti-infection is indicated as: +++, extensive; ++, significant; +, evidence present; -, evidence absent*.

### Partial Purification of Allspice

**Figure 7:**
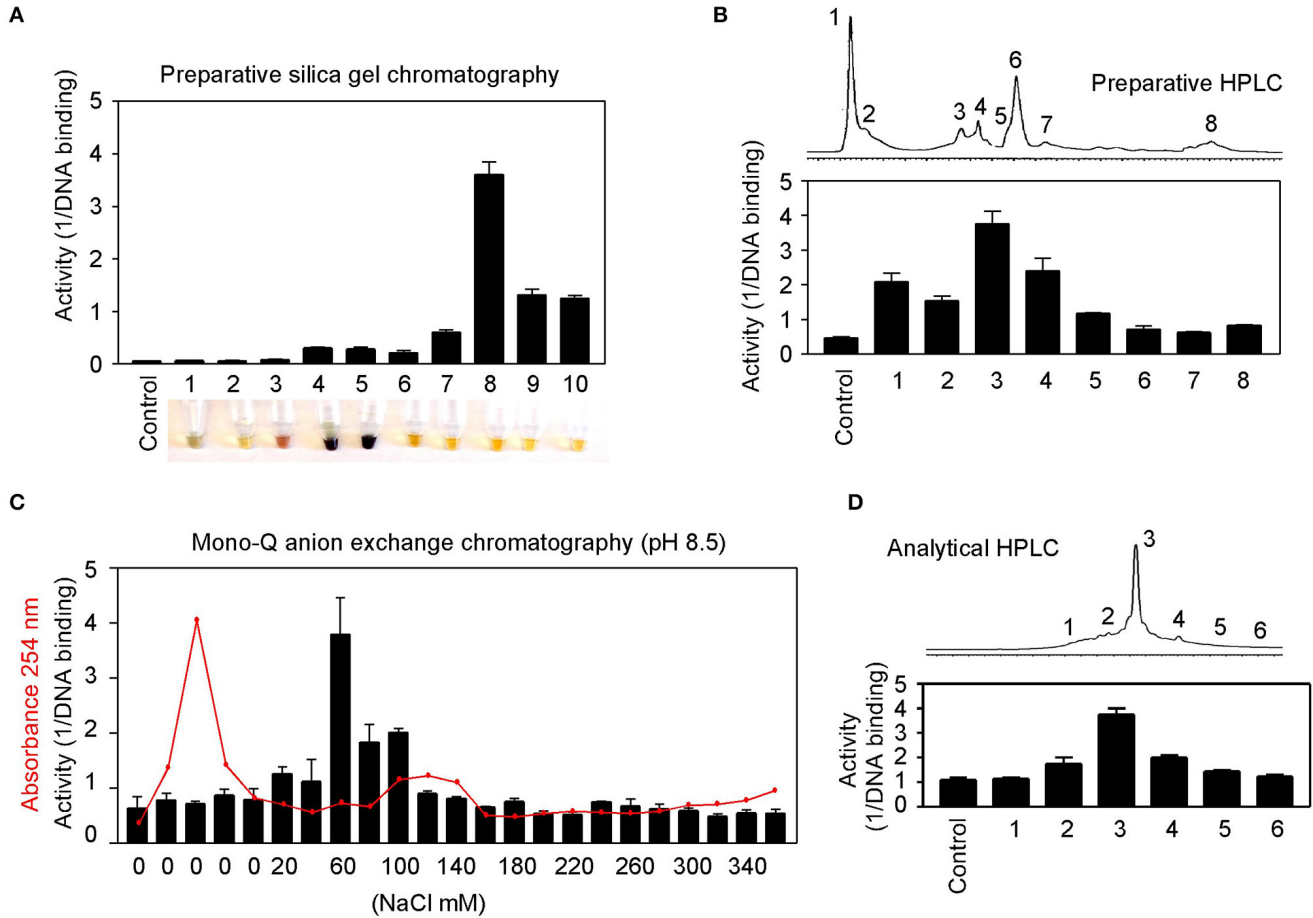
Partial purification of allspice. **(A)** Ethyl acetate extract of allspice was purified by silica gel chromatography to obtain an active fraction 8. Colors of each fraction are shown below. **(B)** Fraction 8 was purified by reverse-phase HPLC to obtain an active peak 3. **(C)** The peak 3 was further purified by mono-Q anion exchange chromatography with active fraction in 60 mM NaCl. **(D)** The 60 mM fraction was analyzed by reverse-phase HPLC with each fraction assayed for activities.

Natural product purification using organic chemical methods requires an efficient *in vitro* assay to monitor constituent activities in each purification step. The highly sensitive DNA-binding assay described above is found to be ideal to monitor purifications of herbs. Allspice extract was first fractionated using silica gel chromatography to obtain an active constituent of fraction 8 (Figure 7A), which was further fractionated by preparative reverse-phase HPLC to obtain an active peak 3 (Figure 7B). The peak 3 was then purified by mono-Q anion exchange chromatography. The most active fraction was eluted at 60 mM NaCl (Figure 7C). The 60-mM fraction was further analyzed by reverse-phase HPLC, and the constituent fraction 3 with the highest activity corresponded to the peak (Figure 7D). Based on further analysis, it appeared that the fraction 3 was not yet pure, and further purification is needed to obtain a single compound.

These results demonstrate great feasibility of natural product purification when constituent activity is monitored by the DNA-binding binding assay using GT198 as the drug target. Because the assay is fast and highly sensitive, we expect that many positive herbs could be purified to obtain chemical drugs or partially purified to remove toxic components to become safer herbal medicines.

## Discussion

GT198 cDNA was first reported in 1995 in an effort to find breast cancer genes in the chromosome 17q21 locus [53]. *GT198* has now emerged out of the shadow of *BRCA1* as an ever-important cancer gene. The original gene symbol at NCBI was *HUMGT198A*, and much later, it was renamed *PSMC3IP*. The full-length human GT198 was initially reported as a transcriptional coactivator [23], and its mouse homolog as a TBP-interacting protein [54]. Subsequently, it was published using the alias name Hop2 in meiosis [55] and in DNA repair [24, 39] due to its functional similarities to yeast Hop2 protein. Today, the alias names in the literature include GT198 in cancer studies, Hop2 or TBPIP in biochemical studies, and *PSMC3IP* in genetic studies.

The divergent research focuses reflect highly complex functions of GT198, which are hard to reconcile during early discoveries. As evidence accumulates, the roles of GT198 become more unified. Regarding the nuclear biochemical activities, GT198 binds to DNA so that it can stimulate transcription, recombination, DNA repair, and meiosis [56]. Many nuclear proteins interact with GT198, including steroid hormone receptors [23] and DNA repair factors [56]. From a cancer biology prospective, GT198 regulates stem cells, stimulates angiogenesis [34, 36], and induces apoptosis [33]. From a genetic prospective, the *GT198* gene carries germlime and somatic mutations in cancers [27, 28, 34] and in ovarian diseases [29]. Our current study extends the role of GT198 into a new dimension as a multidrug target, providing further support for GT198 in oncogenesis [57].

The finding of multiple clinical successful oncology drugs targeting GT198 (Table 1), invites a revisit of previous mechanisms of the action of many drugs. We found that much evidence in the past is indeed consistent with a mechanism targeting to GT198. The GT198 protein is homologous to DNA topoisomerases I and II (Figure 3E), providing a direct explanation of doxorubicin, etoposide, and camptothecin and their homologs as GT198 inhibitors. Consistently, suppression of angiogenesis has also been shown in doxorubicin [58], etoposide [59], and camptothecin [60]. The clinical cardiovascular side effects of doxorubicin may also be correlated with the inhibition of blood vessel pericytes. Dactinomycin is cytotoxic and regulates DNA functions [61], which can be explained as a GT198 inhibitor. In addition, the mechanism of paclitaxel has been linked to mitotic arrest, apoptosis, and angiogenesis [62], and paclitaxel's clinical side effects are correlated with the normal expression of GT198 [10, 45]. Consistent with roles as angiogenesis inhibitors, paclitaxel and docetaxel are common chemotherapy drugs used for human oral cancer. Inhibition of angiogenesis is also shown as an activity in Gleevec [63], aminolevulinic acid [64], and celastrol [65]. Platinum DNA inhibitors did not inhibit GT198 directly (Figure 2D and Table 1), possibly due to their DNA crosslinking rather than DNA intercalating property. The above evidence collectively supports GT198 as a master oncoprotein unmatched by many others characterized to date. Potentially, more GT198 inhibitors could be found in the future because many drugs have not been tested.

Herbs have been a principal form of medicine since ancient times and are still used in most developing countries. Mounting sophisticated uses of herbs have been described in numerous medicinal books, and around 70,000 plant species have been used as medicines throughout history [66]. Because humans co-evolved together with plants in the environment, many natural ingredients have been adapted by humans with less toxicity when compared to chemically synthesized non-natural compounds. Various successful clinical oncology drugs are originally derived from plants, such as paclitaxel from the Pacific yew tree and etoposide from Mayapple.

After our discovery of GT198 inhibitors among existing oncology drugs and realizing that none of them are perfect in both GT198 binding affinity and efficacy, we speculated that better GT198 inhibitors potentially exist in anticancer herbs. This judgment was based on the fact that GT198 plays an important role in cancer initiation, GT198 protein is a druggable target, and mounting historical testimonies are present in anticancer herbs. We then selected and tested herbal medicines with successful human cancer treatment.

GSL (*Gleditsia sinensis L*.) was selected from a Chinese medicinal book named Ben Cao Gang Mu, a UNESCO registered heritage, in which the texts were adapted from an ancient herbal scientist Li Shi-Zhen (1518–1593) [67]. Among more than 1200 herbs described, only GSL stands out to have an impact on all sex organs, including breast, ovary, testis, and uterus, which link to functional characteristics of GT198. GSL is described by the book as super effective to treat women's abdominal tumor masses. Today, GSL is widely used as an anticancer herb in Asia with the tree massively planted for therapeutic interests. Approximately 100 references in PubMed describe the studies of *Gleditsia sinensis*, including anticancer activities in breast and prostate cancers [68, 69] and in tumor angiogenesis [70, 71]. GSL biological activity is also previously summarized [72].

The selection of allspice and BIRM was due to their historical treatments of human prostate cancers in South America. Allspice is well-known for its health benefits, including anti-infection, antioxidant, and enhancing immune properties. Allspice is often used by dentists for oral antiseptic healthcare. Researchers from our own institute showed that active ingredients exist in allspice extract [49, 73, 74], and in BIRM [50, 51] with apoptotic and anticancer activities in mouse models. Both herbs affect androgen receptor–mediated functions in prostate cancer. Because GT198 is an androgen receptor coactivator, we tested and confirmed GT198 as a direct target (Figures 5A, 6C).

An herbal remedy from Taiwan containing branches of four trees was selected to test because this medicine has extensive testimonies in the treatment of human colon cancer. Mulberry and walnut have been described by medicinal books with various health benefits, and island mahonia and rosewood are well-known anticancer herbs with a long history. Although they showed less activity than allspice in inhibition of GT198 (Figure 6A), the combination of the four may have a synergistic anticancer effect. The advantage is that all four tree herbs are less toxic.

In addition, we concurrently tested many other herbal extracts as negative controls (Table 2 and data not shown) to ensure detected activities are not nonspecific due to isolating from plants. The highest sensitivity was observed in GSL extract, which can be detected at 0.2 ng/μl. Given more than hundreds of different molecules that are normally present in the crude extracts, the active compound affinity would predictably be extremely high (IC_50_ <1 nM) if GSL were purified. When toxicity is considered, allspice may be a best medicine without complete purification because it is a common organic spice with plenty of health benefits.

After anti-GT198 activity is found in herbal medicines, an interesting phenomenon has emerged that all positive anticancer herbs are also anti-infection (Table 2). In fact, allspice and BIRM are more prominent for their antiviral and antibacterial activities than for anticancer activity. GSL and rosewood have been used in combating the COVID-19 pandemic in 2020. This is not surprising if considering the fact that acute inflammation in infection and chronic inflammation in cancer share the same angiogenic pericytes, in both cases overexpressing GT198. Inflammatory signals normally aim to activate pericyte stem cells for subsequent growth and inflammatory responses except that chronic and persistent pericyte activation leads to cancer. Thus, a subset of drugs as GT198 inhibitors will likely be both anti-infective and anticancer. The reason most oncology drugs have limited uses is because the drugs are only approved within the scope of conducted clinical trials in a subset of cancer patients. Our current finding helps to expand the usage of approved oncology drugs (Table 1) in angiogenic cancers, including oral cancer as well as in infectious diseases. The effort may accelerate combating future pandemics, not only using herbal medicines, but also using approved chemical drugs. GT198 is essential for the development of dual-effective anticancer and anti-infective drugs in the future.

In summary, we extend the previous observations and further showed that angiogenic pericytes express GT198 in human oral cancer. We identified a panel of existing oncology drugs, including mitoxantrone, doxorubicin, paclitaxel, etoposide, dactinomycin, and imatinib, as direct GT198 inhibitors. We further found a number of anticancer herbs with historical success in human cancer treatment as GT198 inhibitors. We confirmed the feasibility of natural product purification in organic chemistry monitored by the GT198 target. Together, this study reveals GT198 as a new targeting mechanism for many existing oncology drugs. GT198 is an excellent drug target suitable for compound identification and natural product purification. This study may also accelerate identification of high-efficacy and low-toxicity drugs to combat both cancer and infection.

## Materials and Methods

### Immunohistochemistry

Polyclonal rabbit antibody against GT198 was affinity purified and previously described [23, 34]. Formalin-fixed paraffin-embedded (FFPE) sections were deparaffinized and dehydrated through xylene and ethanol series, followed by antigen retrieval in 10 mM sodium citrate buffer, pH 6.0, containing 0.05% Triton at 90°C for 20 min. Anti-GT198 (1:200) was incubated at 4°C overnight. Antibody binding was detected using biotinylated secondary antibody followed by detecting reagents (Abcam). Sections were counterstained with hematoxylin.

Human oral tumor FFPE sections were obtained from the Head and Neck Cancer SPORE at the University of Pittsburgh Cancer Institute. Samples were obtained following institutional IRB guidelines using de-identified human cancer specimens that cannot be traced back to the subject. Clinical staging of five oral cancer specimens used in this study were (1) male, age 56, normal oral mucosa, Figure 1B, quiescent vessel; (2) male, age 50, T4N3M0, Figure 1B, angiogenic vessel; (3) male, age 53, T3N2M0, Figure 1B, detached pericytes; (4) male, age 65, T2N1M0, Figure 1B, vessel in tumor; and (5) male, age 51, T3N1M0, adjacent tissue in Figure 1C; tumor tissue in Figure 1D and in Supplementary Figure 1.

### Oncology Drugs

The Approved Oncology Drugs Set VII containing 129 drugs (plates 4,845 and 4,846) were derived from NCI, Division of Cancer Treatment and Diagnosis (DCTD), and Developmental Therapeutics Program (DTP). Chemical structures of each drug compound can be found in the NCI's website https://dtp.cancer.gov/dtpstandard/platemap/index.jsp, and the drugs are also listed in Supplemental Table 1. Each well in the 96-well plates contained 20 μl of 10 mM drugs in DMSO. In addition to 129 NCI drugs, a number of clinical oncology drugs were obtained from the Augusta University pharmacy.

### Herbal Materials

The GSL herb with the Chinese name ZaoJiaoCi is the thorn needles of *Gleditsia sinensis L*. tree from Yunnan province in China. Its ethanol extract dry powder was provided by the Drug Research Institute of KPC Pharmaceuticals Inc. in Yunnan. Allspice with an origin of Jamaica was obtained from the World Spice Merchants, WA, USA. The dry powder of BIRM extract originated from Ecuador and was provided by Dr. Bal Lokeshwar at Augusta University. The branches of four trees in an anticancer herbal mix containing mulberry, walnut, island mahonia, and rosewood were from an herbal drug store in Taiwan. Two negative control herbs, licorice and garden mum, were obtained from a TongRenTang herbal retail store in China (Table 2). Ethanol extracts of herbs were used in the DNA-binding assays to determine activities. Methanol extracts of GSL and allspice were used in chromatography purifications. The extracts were dried overnight, weighted using an analytical balance, dissolved in DMSO at 30 mg/ml, and stored at −80°C.

### His-Tagged GT198 Recombinant Protein Purification

The full-length GT198 protein has 217 amino acids [23]. The N-terminus and the DNA-binding domain are essential for its dimerization and DNA binding. However, we have previously reported that its C-terminal tail (aa 181–217) reduces its own DNA-binding activity and serves as an auto-inhibitory domain [33]. *In vivo* GT198 activity might be tightly guarded until regulated. For *in vitro* assays, the C-terminal removal will enable maximal DNA binding and signal detection. Thus, this study used a C-terminal truncated version of GT198 (aa 1–180) to ensure high sensitivity of the assay. The potential drawback of the C-terminal removal is that certain drugs may not be detectable if the C terminus is required.

N-terminal His-tagged recombinant human GT198 protein without its C-terminus (aa 1–180) were expressed in *E. coli* BL21 (DE3) pLysS and purified through Ni-NTA-agarose (Qiagen) as previously described [33]. Proteins were eluted by 200 mM imidazole, desalted, concentrated using Amicon YM-10 spin columns, and stored at −80°C before use. Protein concentrations were determined using protein assay dye reagent concentrate solution (Bio-Rad) and 2 mg/ml bovine serum albumin (BSA) standard (Sigma).

### DNA Binding and Competition Assays

In the *in vitro* 96-well plate binding assay, the binding of biotinylated DNA to GT198 was detected by chemiluminescence. A single-stranded 25-mer biotin-oligonucleotide (Biotin)-cctggggttgctgaggtcctggcag was used in the assay because it is sufficient to bind one GT198 dimer. White MicroLite^TM^ 2+ 96-well plates (Thermo Scientific, #7572) were coated by drying overnight at 37°C containing a solution of 400 ng/well of recombinant His-tagged GT198 proteins together with 5 ug/well of purified BSA (NEB) in a volume of 50 ul. BSA alone was included as a control for background. No subtraction of background was needed due to very low background. Duplicated wells were used for each experimental point. Each experiment was repeated three times. The GT198-coated plates were blocked with 5% BSA in a solution containing PBS with 0.1% Triton X-100 (TPBS) for 1 h. The binding was carried out for 4 h to overnight at 4°C using 150 nM Biotin-DNA and serial diluted drugs (0.128, 0.64, 3.2, 16, 80, 400, 2,000, 10,000 nM) or herb extracts (0, 0.0384, 0.192, 0.96, 4.8, 24, 120, 600 ng/μl) in the binding buffer (20 mM TrisHCl, pH 7.5, 50 mM NaCl, 75 mM KCl, 0.5 mM MgCl_2_, 0.05% Triton X-100, 10% glycerol, 1 mM dithiothreitol). After binding, the plates were washed three times with TPBS solution for total 45 min. The plates were then incubated with streptavidin-conjugated HRP (Roche Molecular Biochemicals, #1089153) at 1 U/ml in TPBS for 1 h at 4°C and further washed three times with TPBS for total 30 min. Bound biotinylated DNA were detected by chemiluminescence with 50 μl/well ECL detection reagents (Amersham Pharmacia Biotech) using a luminometer. Reading of plates was repeated three times within 45 min, and usually the second reading yielded the most consistent results. The finished plates were stained with Bio-Rad protein assay dye reagent to evaluate the bound GT198 protein on the plates. MicroLite^TM^ 2+ plates have excellent binding capacity without losing coated GT198 protein after repeated washing steps.

### Kinetic Data Analysis

The IC_50_ value is the drug concentration of 50% inhibition of the DNA binding. The IC_50_ values were calculated through Kaleidagraph software (Synergy Software) using nonlinear regression sigmoidal dose-response curve fit. The equation for calculation is y = m1 + (m2 – m1)/[1 + (x /m3)^m4^], where m1 is the minimum, m2 is the maximum, m3 is the IC_50_ value calculated by Kaleidagraph, and m4 is the slope at midpoint of the curve. The analysis of Vmax and Km in competitive binding assays were carried out in double-reciprocal plots, using means of four high-concentration data points, omitting points at lower concentrations with reciprocal values off-scale. See a reference as examples [45]. Double-reciprocal plots reveal competitive binding as constant Vmax with increased Km in the presence of drugs or allosteric binding with reduced Vmax and constant Km in the presence of drugs. In general, competitive inhibitors directly bind to the DNA-binding surface of the target and kick out the DNA. By contrast, allosteric inhibitors bind to a site nearby and induce protein conformational changes to reduce the DNA-binding to the target. Competitive inhibitors may have higher binding efficacy.

### Polarity of Herbal Ingredient

Silica gel is highly polar and can interact strongly with compounds. Using a gradient of nonpolar to polar solvents, compounds with different polarity can be separately eluted from silica gel in constituents. The 5 ml methanol extracts containing 60 mg of GSL or allspice were mixed with 1.5 g of high-purity grade, pore size 60 Å, 70–230 mesh silica gel (Sigma #288624), and air-dried overnight. The herb-bound silica gel was loaded on to a chromatography column (Bio-Rad, 3 ml gel in 14 ml column) and batch eluted by 6 ml of the following solvents with polar index in parentheses. Hexane (0.1), 1:1 hexane and ethyl acetate (2.2), 1:2 hexane and ethyl acetate (3.0), ethyl acetate (4.4), 1:1 ethyl acetate and methanol (4.7), methanol (5.1), and 1:1 methanol and water (7.6). The eluted fractions were vacuum dried, weighted, and tested for activity in the DNA-binding assays.

### Organic Compound Purification of Allspice

Allspice berries (50 g) were ground into powder and extracted by 1,000 ml methanol overnight at room temperature. The methanol-soluble fraction was liquid–liquid extracted using an equal volume of ethyl acetate. The ethyl acetate fraction was further dried for subsequent chromatography. The extracted allspice (1.5 g) was bound to silica gel and loaded onto a silica gel column (3 × 30 cm), which was first eluted by hexane-chloroform gradient and then by ethyl acetate-10% methanol in ethyl acetate gradient. A total of 10 fractions (50 ml each) were collected, and the highest activity was in fraction 8 (Figure 7A). This fraction was concentrated and further purified by preparative reverse-phase HPLC (C18, 4.6 × 150 mm, flow rate 0.5 ml/min), using 5% methanol in water to 100% methanol gradient to obtain an active peak 3 (Figure 7B). The peak three was dried (600 μg) and further purified by mono-Q anion exchange chromatography (Bio-Rad HiTrap Q HP column, 1 ml). The column was step-eluted by 1.2 ml of increasing concentration of NaCl (0–360 mM) in a buffer containing 95% methanol, 50 mM NH_4_HCO_3_, pH 8.5. All fractions were measured for absorbance at A_254_ and assayed for activity. The most active fraction was eluted at 60 mM NaCl (Figure 7C). The 60 mM fraction was then analyzed by reverse-phase HPLC (Waters, C18, 4.6 × 75 mm, flowrate 0.5 ml/min), yielding a fraction 3 with highest activity (Figure 7D). In each step, the testing samples were dried and dissolved in DMSO or in the binding butter for the DNA-binding assays.

### Apoptosis Tunel Assay

HeLa cells (ATCC, CCL-2) were maintained in chamber slides in DMEM supplemented with 10% fetal bovine serum, 100 U/ml penicillin, and 0.1 μg/μl streptomycin. Cells were incubated in 5% CO_2_ at 37°C. Cells were treated with 200 ng/μl GSL extract overnight and fixed with 100% methanol for 10 min at −20°C. Cells were then made permeable using 0.1% Triton in 10 mM sodium citrate pH 6.0 for 2 min on ice. TUNEL staining was carried out using the *In Situ* Cell Death Detection Kit (Roche) following the manufacturer's protocol. Briefly, prepared cells in a chamber side were labeled in the dark for 1 h at 37°C with TMR-red labeled dUTP and terminal transferase solution, which labels DNA fragments produced in apoptotic cells. After washing with PBS, slides were counterstained with DAPI before visualization by fluorescence microscopy.

### Protein Sequence Alignment

Protein sequence alignment was carried out using the Multiple Sequence Alignment tool of Clustal Omega at EMBL-EBI (https://www.ebi.ac.uk/Tools/msa/clustalo/). Protein sequences of human GT198, human DNA topoisomerase I, and IIB were entered for alignment using default settings in the program. Asterisks denote identical amino acid residues, and dots denote homologous residues (Supplementary Figure 2). Selected section of results was shown in Figure 3E.

### Statistical Analysis

Statistical analyses were carried out using GraphPad Prism software. Bar graphs were derived from data points in duplicates and are presented for the DNA-binding assays. *P*-values were calculated using unpaired two-tailed *t*-test. ^*^P < 0.05, ^**^P < 0.01, ^***^P < 0.001; NS, not significant. A *P-*value of < 0.05 is considered statistically significant.

## Data Availability Statement

The original contributions presented in the study are included in the article/Supplementary Material, further inquiries can be directed to the corresponding authors.

## Author Contributions

LK and JG conceived and designed experiments. LK, JP, JG, and LZ performed experiments. JP, LZ, and NM contributed reagents. LK wrote manuscript. All authors contributed to the article and approved the submitted version.

## Conflict of Interest

LK is a founder of OnkoTarget LLC, and is the inventor of GT198 patents. The remaining authors declare that the research was conducted in the absence of any commercial or financial relationships that could be construed as a potential conflict of interest.
