# Supplementary material for: GT198 Is a Target of Oncology Drugs and Anticancer Herbs
Source: Front Oral Health. 2021 Jun 11;2:679460. doi: 10.3389/froh.2021.679460 (PMC8409151; doi:10.3389/froh.2021.679460)
Supplement: Supplementary file 1 [file Data_Sheet_1.PDF]

Supplementary Material

Article Title: GT198 Is a Target of Oncology Drugs and Anticancer Herbs

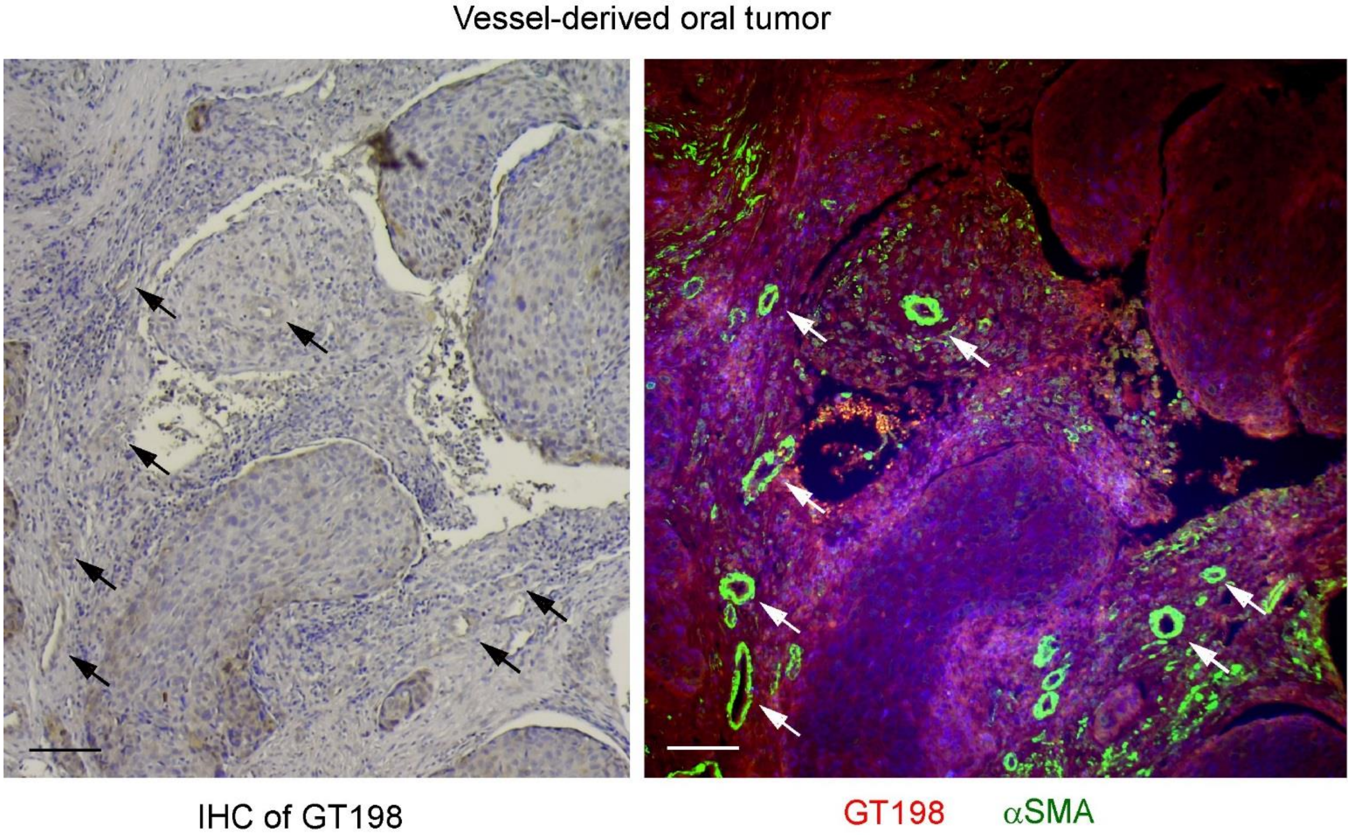

Supplementary Figure 1. Angiogenic blood vessel-derived human oral tumor. Extended view of Figure 1D. Adjacent human oral tumor sections were immunohistochemical stained with GT198 (left panel), and fluorescent doubled stained with GT198 in red and αSMA in green (right panel). Arrows indicate $GT198^+$ and αSMA^+ blood vessels. Angiogenic vessels are initially located in the stroma. When tumors develop surrounding the vessels, blood vessels disintegrate and αSMA^+ cells become scattered into the new growth of tumor. In contrast, the advanced tumors have a few diluted αSMA^+ cells (lower center and upper right areas). Scale bars = 100 μm.

Clinical staging of human oral cancer FFPE specimens:

1) Male, age 56, Normal oral mucosa, Figure 1B, quiescent vessel.
2) Male, age 50, T4N3M0, Figure 1B, angiogenic vessel.
3) Male, age 53, T3N2M0, Figure 1B, detached pericytes.
4) Male, age 65, T2N1M0, Figure 1B, vessel in tumor.
5) Male, age 51, T3N1M0, adjacent tissue in Figure 1C; tumor in Figure 1D and in Supplementary Figure 1.

Supplementary Figure 2. Protein sequence alignment of human GT198, human DNA topoisomerase I (Top1), and IIB (Top2B) using Clustal Omega at EMBL-EBI. Asterisks denote identical amino acid residues and dots denote homologous residues.

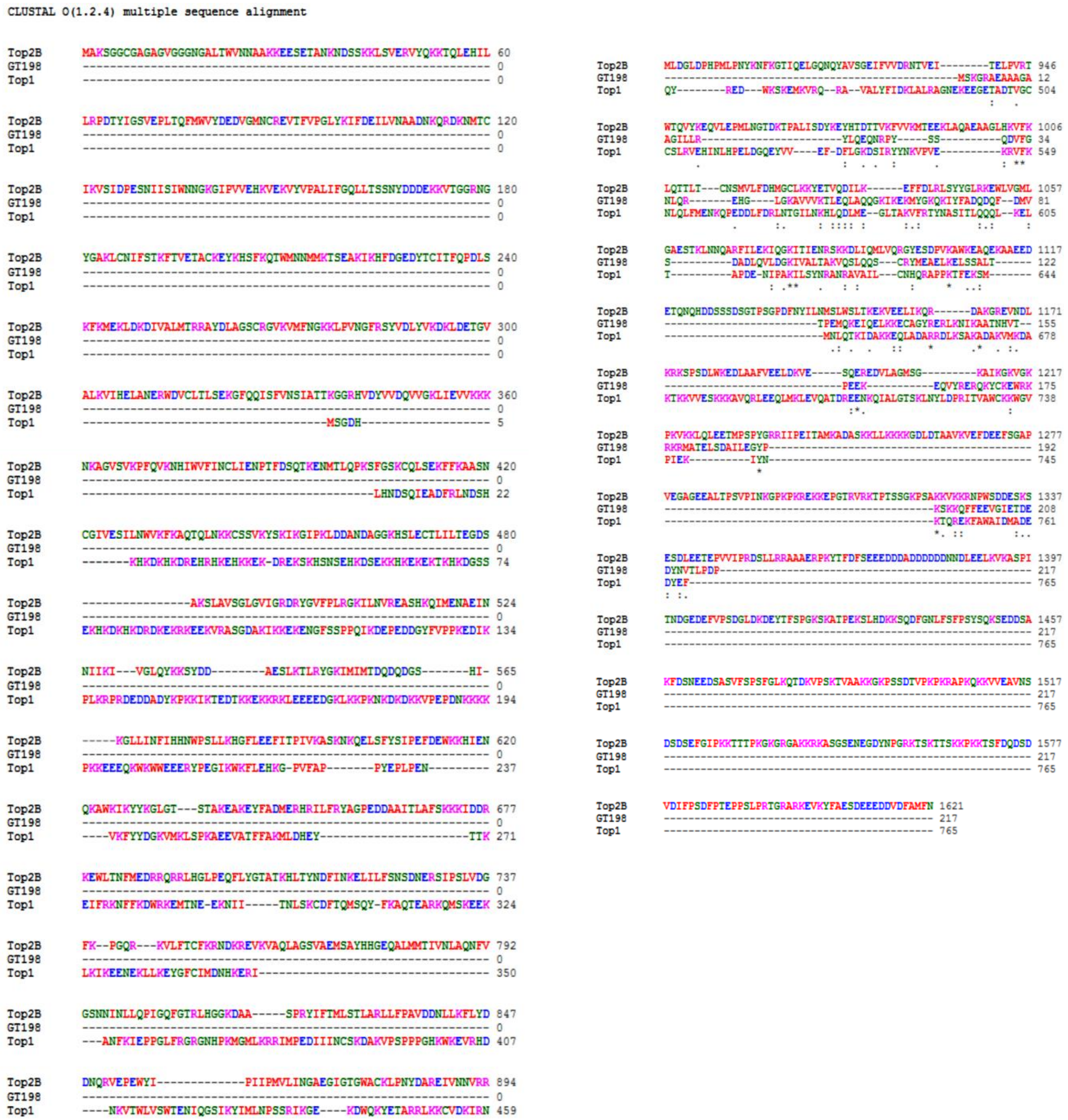

Supplementary Table 1. Identification of GT198 inhibitors from 129 clinical oncology drugs. The FDA Approved Oncology Drug Set VII from NCI, Plate 4845 and Plate 4846, were tested using the DNA-binding assay for direct inhibition of GT198. Available IC_{50} values and efficacies (percent of inhibition at 2 μM of drugs) are shown. -, not active; +, active; ND, not detectable.

Inhibition of GT198 (NCI Approved Oncology Drug Set VII)								
Plate	Well ID	Compound ID	CAS Number	Drug Name	MW	Activity	IC50	Efficacy (2 μM)
4845	A02	1390	315-30-0	Allopurinol	136.11	—		
4845	B02	19893	51-21-8	Fluorouracil	130.08	—		
4845	C02	32065	127-07-1	Hydroxyurea	76.05	—		
4845	D02	752	154-42-7	Thioguanine	167.19	—		
4845	E02	755	50-44-2	Mercaptopurine	152.18	—		
4845	F02	762	55-86-7	Mechlorethamine	192.52	—		
4845	G02	6396	52-24-4	Thiotepa	189.22	—		
4845	H02	18509	5451-09-2	Aminolevulinic acid	167.59	+		
4845	A03	45388	4342-03-4	Dacarbazine	182.18	—		
4845	B03	362856	85622-93-1	Temozolomide	194.15	—		
4845	C03	750	55-98-1	Busulfan	246.30	—		
4845	D03	13875	645-05-6	Altretamine	210.28	—		
4845	E03	27640	50-91-9	Floxuridine	246.19	—		
4845	F03	45923	298-81-7	Methoxsalen	216.19	—		
4845	G03	79037	13010-47-4	Lomustine	233.70	—		
4845	H03	102816	320-67-2	Azacitidine	244.21	—		
4845	A04	127716	2353-33-5	Decitabine	228.21	—		
4845	B04	296961	20537-88-6	Amifostine	214.22	—		
4845	C04	409962	154-93-8	Carmustine	214.05	—		
4845	D04	26271	6055-19-2	Cyclophosphamide	261.09	—		
4845	E04	34462	66-75-1	Uracil mustard	252.10	—		
4845	F04	63878	69-74-9	Cytarabine	279.70	—		
4845	G04	66847	50-35-1	Thalidomide	258.23	—		
4845	H04	75520	70-00-8	Trifluridine	296.20	+		
4845	A05	77213	366-70-1	Procarbazine	257.76	—		
4845	B05	85998	18883-66-4	Streptozocin	265.22	—		
4845	C05	105014	4291-63-8	Cladribine	285.69	—		
4845	D05	109724	3778-73-2	Ifosfamide	261.09	—		
4845	E05	119875	15663-27-1	Cisplatin	300.06	—		
4845	F05	122758	302-79-4	Tretinoin	300.44	—		
4845	G05	169780	24584-09-6	Dexrazoxane	268.27	—		
4845	H05	218321	53910-25-1	Pentostatin	268.27	—		
4845	A06	613327	122111-03-9	Gemcitabine	299.65	—		
4845	B06	701852	149647-78-9	Vorinostat	264.32	—		
4845	C06	713563	107868-30-4	Exemestane	296.40	—		
4845	D06	719344	120511-73-1	Anastrozole	293.37	—		
4845	E06	719345	112809-51-5	Letrozole	285.30	—		
4845	F06	721517	118072-93-8	Zoledronic acid	272.09	—		

4845	G06	747972	191732-72-6	Lenalidomide	259.26	–		
4845	H06	755985	121032-29-9	Nelarabine	297.27	–		
4845	A07	775351	19171-19-8	Pomalidomide	273.25	–		
4845	B07	3088	305-03-3	Chlorambucil	304.22	–		
4845	C07	26980	50-07-7	Mitomycin	334.33	–		
4845	D07	38721	53-19-0	Mitotane	320.04	–		
4845	E07	606869	123318-82-1	Clofarabine	303.68	–		
4845	F07	758774	414864-00-9	Belinostat	318.35	–		
4845	G07	761190	404950-80-7	Panobinostat	349.43	–		
4845	H07	25154	54-91-1	Pipobroman	356.06	–		
4845	A08	71423	595-33-5	Megestrol acetate	384.51	–		
4845	B08	138783	3543-75-7	Bendamustine	394.73	–		
4845	C08	241240	41575-94-4	Carboplatin	371.25	–	ND	0%
4845	D08	266046	61825-94-3	Oxaliplatin	397.29	–		
4845	E08	312887	75607-67-9	Fludarabine	365.21	–		
4845	F08	712807	154361-50-9	Capecitabine	359.35	–		
4845	G08	719627	169590-42-5	Celecoxib	381.37	–		
4845	H08	750690	557795-19-4	Sunitinib	398.47	+		
4845	A09	756655	179324-69-7	Bortezomib	384.24	–		
4845	B09	757441	319460-85-0	Axitinib	386.47	–		
4845	C09	279836	65271-80-9	Mitoxantrone	444.49	+	187 nM	90%
4845	D09	715055	184475-35-2	Gefitinib	446.90	–		
4845	E09	718781	183319-69-9	Erlotinib	429.90	–		
4845	F09	753686	763113-22-0	Olaparib	434.46	–		
4845	G09	755980	417716-92-8	Lenvatinib	426.86	–		
4845	H09	755986	879085-55-9	Vismodegib	421.30	–		
4845	A10	756645	877399-52-5	Crizotinib	450.34	–		
4845	B10	759224	870281-82-6	Idelalisib	415.42	–		
4845	C10	761910	936563-96-1	Ibrutinib	440.50	–		
4845	D10	740	59-05-2	Methotrexate	454.44	–		
4845	E10	609699	119413-54-6	Topotecan	457.91	–	>2 μM	4%
4845	F10	732517	863127-77-9	Dasatinib	488.01	–		
4845	G10	737754	635702-64-6	Pazopanib	473.98	–		
4845	H10	743414	152459-95-5	Imatinib	493.61	+		
4845	A11	747971	284461-73-0	Sorafenib	464.82	–		
4845	B11	747974	84449-90-1	Raloxifene	473.59	–		
4845	C11	750691	439081-18-2	Afatinib	485.94	–		
4845	D11	754230	146464-95-1	Pralatrexate	477.48	–		
4845	E11	755384	357166-30-4	Pemetrexed	471.38	–		
4845	F11	755605	915087-33-1	Enzalutamide	464.42	–		
4845	G11	760766	443913-73-3	Vandetanib	475.36	–		
4845	H11	761385	956697-53-3	Erismodegib	485.49	–		

4845	G06	747972	191732-72-6	Lenalidomide	259.26	—		
4845	H06	755985	121032-29-9	Nelarabine	297.27	—		
4845	A07	775351	19171-19-8	Pomalidomide	273.25	—		
4845	B07	3088	305-03-3	Chlorambucil	304.22	—		
4845	C07	26980	50-07-7	Mitomycin	334.33	—		
4845	D07	38721	53-19-0	Mitotane	320.04	—		
4845	E07	606869	123318-82-1	Clofarabine	303.68	—		
4845	F07	758774	414864-00-9	Belinostat	318.35	—		
4845	G07	761190	404950-80-7	Panobinostat	349.43	—		
4845	H07	25154	54-91-1	Pipobroman	356.06	—		
4845	A08	71423	595-33-5	Megestrol acetate	384.51	—		
4845	B08	138783	3543-75-7	Bendamustine	394.73	—		
4845	C08	241240	41575-94-4	Carboplatin	371.25	—	ND	0%
4845	D08	266046	61825-94-3	Oxaliplatin	397.29	—		
4845	E08	312887	75607-67-9	Fludarabine	365.21	—		
4845	F08	712807	154361-50-9	Capecitabine	359.35	—		
4845	G08	719627	169590-42-5	Celecoxib	381.37	—		
4845	H08	750690	557795-19-4	Sunitinib	398.47	+		
4845	A09	756655	179324-69-7	Bortezomib	384.24	—		
4845	B09	757441	319460-85-0	Axitinib	386.47	—		
4845	C09	279836	65271-80-9	Mitoxantrone	444.49	+	187 nM	90%
4845	D09	715055	184475-35-2	Gefitinib	446.90	—		
4845	E09	718781	183319-69-9	Erlotinib	429.90	—		
4845	F09	753686	763113-22-0	Olaparib	434.46	—		
4845	G09	755980	417716-92-8	Lenvatinib	426.86	—		
4845	H09	755986	879085-55-9	Vismodegib	421.30	—		
4845	A10	756645	877399-52-5	Crizotinib	450.34	—		
4845	B10	759224	870281-82-6	Idelalisib	415.42	—		
4845	C10	761910	936563-96-1	Ibrutinib	440.50	—		
4845	D10	740	59-05-2	Methotrexate	454.44	—		
4845	E10	609699	119413-54-6	Topotecan	457.91	—	>2 μM	4%
4845	F10	732517	863127-77-9	Dasatinib	488.01	—		
4845	G10	737754	635702-64-6	Pazopanib	473.98	—		
4845	H10	743414	152459-95-5	Imatinib	493.61	+		
4845	A11	747971	284461-73-0	Sorafenib	464.82	—		
4845	B11	747974	84449-90-1	Raloxifene	473.59	—		
4845	C11	750691	439081-18-2	Afatinib	485.94	—		
4845	D11	754230	146464-95-1	Pralatrexate	477.48	—		
4845	E11	755384	357166-30-4	Pemetrexed	471.38	—		
4845	F11	755605	915087-33-1	Enzalutamide	464.42	—		
4845	G11	760766	443913-73-3	Vandetanib	475.36	—		
4845	H11	761385	956697-53-3	Erismodegib	485.49	—		
